# Musicality in human vocal communication: an evolutionary perspective

**DOI:** 10.1098/rstb.2020.0391

**Published:** 2022-01-03

**Authors:** Juan David Leongómez, Jan Havlíček, S. Craig Roberts

**Affiliations:** ^1^ Human Behaviour Lab, Faculty of Psychology, Universidad El Bosque, Bogota, Colombia; ^2^ Department of Zoology, Charles University, Prague, Czech Republic; ^3^ Faculty of Natural Sciences, University of Stirling, Stirling, UK

**Keywords:** musicality, music, acoustic communication, voice modulation, infant-directed speech, evolution

## Abstract

Studies show that specific vocal modulations, akin to those of infant-directed speech (IDS) and perhaps music, play a role in communicating intentions and mental states during human social interaction. Based on this, we propose a model for the evolution of musicality—the capacity to process musical information—in relation to human vocal communication. We suggest that a complex social environment, with strong social bonds, promoted the appearance of musicality-related abilities. These social bonds were not limited to those between offspring and mothers or other carers, although these may have been especially influential in view of altriciality of human infants. The model can be further tested in other species by comparing levels of sociality and complexity of vocal communication. By integrating several theories, our model presents a radically different view of musicality, not limited to specifically musical scenarios, but one in which this capacity originally evolved to aid parent–infant communication and bonding, and even today plays a role not only in music but also in IDS, as well as in some adult-directed speech contexts.

This article is part of the theme issue ‘Voice modulation: from origin and mechanism to social impact (Part II)’.

## 1. Introduction

Musicologists have commonly rejected the idea of music as a universal phenomenon (e.g. [1–3]; but see [4]), and therefore the study of its origins has not been often addressed within this discipline. By contrast, scientists from disciplines such as biology, psychology and anthropology have long-standing interest in this idea, often focusing on the purpose of music and its potential evolutionary origin. This essential difference, which perhaps exists because ethnomusicologists usually look at cultural differences and focus on the specificity of individual musical manifestations, while researchers from other disciplines see music as a universal, human phenomenon, has often prevented communication between these complementary views.

While it seems undeniable that all cultures have some individual musical manifestations (i.e. any musical output, from singing and instrument-playing to clapping and dancing) that can be recognized as music [5], ethnomusicologists often highlight the colossal variation in their social contexts and meanings. This makes the scientific study of music as a human universal, and understanding of its origins, extremely challenging.

Scientists have often focused on *music*, which is a behavioural manifestation—the outcome of any potential adaptations—rather than the adaptations themselves. In other words, it may be more pertinent to examine *musicality*, our ability to process musical information, than *music*. Additionally, musicality consists of different separable mechanisms for production and perception that may have evolved independently [6]. Furthermore, because most theories for the origin of music point to an evolutionary connection between music and language, the domain of musicality might be not limited to music but might also play a role in infant-directed speech (IDS) [7] and perhaps even in adult-directed language.

In this paper, we first outline the problems posed by the study of the origins of music, including possible human universals and the relationship between language and music. We then review the evidence regarding the evolution of music, addressing the challenges posed by the evolutionary study of music and reviewing the key current ideas on this topic. In so doing, we consider both animal precursors and theories for the evolution of human acoustic communication, as well as their limitations. Finally, we propose a theoretical model for the evolution of musicality, which provides an explanatory and testable framework for explaining newly emerged findings in human acoustic communication. Beyond IDS, recent research has shown that contextual vocal modulation is important (reviewed in the previous issue (part I) by Hughes & Puts [8]). For example, fundamental frequency variability (commonly measured as *f*_0_SD or *f*_0_CV) is modulated and plays an important role in courtship contexts (e.g. [9–11]). However, this key role of *f*_0_ variability is not apparent in non-courtship scenarios, including authority ranking relationships (e.g. [12,13]), perhaps indicating context-specific variation in the importance of musicality in human communication. Such variation could help us to discover and define the evolutionary processes that have shaped the emergence of human musicality.

## 2. The puzzling origins of music

There are clear differences in form and complexity between human and non-human forms of vocal communication, which make the understanding of the evolution of human acoustic communication a huge challenge. For example, Falk [14, p. 491] asked, ‘why are we the only animals that talk?’. Similarly, Brown & Jordania [15, p. 230] noted that, among more than 4500 singing species living today, only humans have ‘the ability to follow precise rhythmic patterns so as to permit group singing, drumming, and dancing’, leading them to ask ‘What explains the unique place of humans among singing species?’.

Finding answers to these questions is made challenging by the lack of clear intermediate stages that allow us to create an accurate picture of the evolutionary process towards modern forms of human acoustic communication. There are no other extant hominin species with varying degrees of acoustic communicative complexity, with which we could compare ourselves. And, as the fossil record does not allow us to directly study the acoustic communication of extinct species, we are forced to rely on indirect inferences based on archaeological findings or observations of modern animal species (including humans). For instance, even in the case of Neanderthals, *Homo neanderthalensis*, probably the most studied extinct human species, there seem to be no clear conclusions about their level of acoustic communication [16,17].

Darwin himself portrayed music as one of the most mysterious human abilities [18]. It is a phenomenon with no obvious function but seems to be present in all human cultures [19], whose roots can be certainly traced for a few tens of thousands of years, back to the earliest known musical instruments. The earliest known flute—a complex instrument capable of producing differentiated pitches—was made around 40 000 years ago [20,21], However, music does not depend for its existence on the construction of instruments, as we can sing, dance and use our bodies or other natural objects as drums and so we can infer that musical ability significantly pre-dates this time.

### (a) The question of music universals

The universality of human music is critical to the study of musicality. If musical production is in fact a universal phenomenon, then the idea of it having a purely cultural origin would be hard to defend, suggesting instead some form of biological antecedent.

This is not to say that culture is unimportant. Unquestionably, cultural distinctiveness and variation define individual features, social roles, meanings and conceptions of music. As Cross [22] points out, within the humanities, including main trends in musicology and ethnomusicology, there is broad consensus in the view of music as a cultural construction. This view is supported precisely by the enormous cultural variation of musical or music-like phenomena in human societies. Moreover, the notion of *music* itself varies significantly between different cultures. For example, Australian Aboriginal songs combine visual, performing and oral arts [23], while the Igbo concept of *nkwa* includes not only actions like singing and playing instruments, but also dancing [24]. In fact, many scholars prefer to use the term *musics* instead of *music*, to account for the uniqueness of these phenomena within each culture (see [2]).

This idea of *musics* as cultural expressions lacking any relevant commonalities, only valid within the context of a particular human group [1,2], is essential to understand the limitations that it places on the study of music. If there are no universals in *musics*—no common basic principles that allow measurements and comparisons to be made—then the scientific study of music as one universal human phenomenon would be irrelevant and perhaps implausible. However, this view is by no means common to all researchers within the humanities (e.g. [5,15,25]); Blacking [5], for example, stated that every society has some cultural manifestations that can be recognized as music, implying that there are, in fact, common features.

In the light of immense cultural variability, it seems difficult to agree about a definition of what music is, and especially how it arose and *what it is for*. However, despite these obstacles, we know that our brain, physiology and psychology render humans capable of producing and listening to music. In other words, we do at least know that the capacity to process musical information, *musicality*, is universal.

For some decades, scientists from diverse disciplines have presented data that speak to us of a more primal, biological basis of musicality, common to all humans. And, furthermore, science has provided an insight into the cognitive demands of musical capacity. We *all* share the amazing capacity to produce, perceive and enjoy—or dislike—music, probably soon after we are born [26–28] (or perhaps even before (e.g. [29,30]), and music has a substantial capacity to affect our emotions [31,32].

In fact, many scientists have proposed a variety of music universals. Fritz *et al*. [33] found that adult Mafa people (from Cameroon and Nigeria) were successful in identifying three basic emotions (happy, sad, scared/fearful) in Western music, at above chance levels. Both Western and Mafa participants also preferred original versions of Western and Mafa music over spectral manipulations of the originals (that affected the sensory dissonance of the music), suggesting that emotionally laden music can be cross-culturally recognized and that its appreciation is universally affected by consonance and dissonance. Trehub [34] proposed the existence of several universal musical features, based on analyses of responses of human infants and adults to original and transposed melodies. These included the perception of contours (i.e. relational pitch and time features of music), scales composed of unequal steps, and a preference for small integer frequency ratios (i.e. consonances, such as the octave (2 : 1), perfect fifth (3 : 2) and perfect fourth (4 : 3)) over large integer ratios (dissonances, such as the tritone (45 : 32)). In addition, Trehub suggested the universality of a music genre for infants (e.g. lullabies and play songs). In fact, adults can recognize a lullaby as such, even when they are unfamiliar with the musical culture, and can identify with almost absolute precision when a song was sung to an infant [35–38].

Brown & Jordania [15] proposed an extensive list of music universals, categorized in four types: (i) *conserved universals*, which apply to all musical utterances and include pseudo-syntactic elements such as music being organized into phrases, the use of relative pitch (RP) elements such as the equivalence of octaves (and consequent transposability of melodies) and the use of discrete pitches, as well as factors used for emotive expression, such as register, tempo and amplitude; (ii) *predominant patterns*, which apply to all musical styles, including rhythmic features such as the predominance of isometric rhythms, the use of scales divided into seven or fewer pitches, the use of motives and use of texts, among others; (iii) *common patterns*, which apply to many styles and include, for example, the association of music and dance, and the use of aerophone instruments (wind instruments); and (iv) *range universals*, which contains a set of possible options for all musical systems, such as textures (monophony, heterophony, homophony or polyphony) and type of arrangement (solo or group arrangements). Many of the musical universals proposed by Brown & Jordania were confirmed empirically by Savage and colleagues through an analysis of 304 recordings of diverse traditional musics from around the world [4].

More recently, and by examining a large sample of societies, Mehr *et al*. [39] have produced evidence showing that musical forms can be described by two dimensions (rhythm and melody), that musical behaviour can be described by three components (arousal, religiosity and formality), and that tonality may be universal. Furthermore, they showed that there are robust cross-cultural associations between form (for example, in dance songs or lullabies) and functions in vocal music (e.g. ‘for dancing’, ‘used to soothe a baby’), and that these functions can be detected by listeners from around the world [36].

### (b) Music–language relationship

Research into universals of musical form and structure suggests that music and its perception is related in complex ways to language, or at least is analogous to it. The deep relationship between language and music in terms of shared neural resources is supported by evidence presented in a variety of studies (e.g. [40–42]) and has become an important area of research and source of debate in recent years.

An increasing number of studies show an important overlap of neural resources involved in the processing of specific music and language tasks (e.g. [43–45]). For instance, strong evidence for shared resources in musical and linguistic syntactic processing has been shown in several studies [40,45]. Moreover, children who suffer from Specific Language Impairment (SLI), which is characterized by deficient processing of linguistic syntax, also show a deficiency of musical syntax processing [46]. There is even evidence suggesting that the human brain does not treat language and music as different kinds of stimuli, at least in early stages of infancy, when it seems to treat language ‘as a special case of music’ [47]. Furthermore, music therapy has been successfully used in speech rehabilitation [48–51].

As in language, music processing involves networks of extensively distributed brain regions. In fact, music might comprise an even vaster network of regions, from both hemispheres, and with an overall asymmetry towards the right hemisphere for pitch processing [52,53]. Hence, the overlap between the activated neural areas for music and language processing that has been found in several neuroimaging studies—especially clear in production tasks that involve singing with lyrics—is not surprising.

Indeed, Peretz [53] points out that in this context—in which overlapping of involved neural resources is expected—finding distinct areas of activation for music and language (particularly singing and speaking) can be more enlightening than describing overlaps. Several studies ([54–59]; for an example of activation in non-singing musical tasks, see [60]) have reported, in addition to the expected overlapping, activation of distinct areas for speech and song production. For example, when singing compared with speaking, there is additional activation on the right superior temporal gyrus and in the primary sensorimotor cortex [58]. Furthermore, evidence of domain-specificity of music and language processing becomes apparent from the study of specific cases of brain damage or developmental disorders [27,53,61,62], in which patients might lose musical abilities while maintaining their speaking capacity, like some amusic patients [27,53,61–63], or when patients can sing or play music, but can no longer speak, as in the case of some aphasias [64,65].

What does this deep relationship tell us about the origins of music and language? Is it possible to think that both channels have common origins? Some genetic evidence seems to suggest that this is precisely the case. For example, Alcock *et al*. [66] found that the *FOXP2* gene—which plays a crucial role in the neural development necessary for language and speech—seems to affect rhythm perception and production, while not affecting pitch perceptual and production skills (which seem to be affected by independent genetic factors, as congenital amusia shows [62]). Furthermore, performance in detecting out-of-key notes in popular melodies showed a stronger correlation between identical (*r* = 0.79) than fraternal (*r* = 0.46) twins, suggesting that genetic influence—with a heritability of 70–80%—is more important than shared environments for musical pitch perception [67,68].

The findings regarding similarities and differences found in the processing of music and language have led to an interesting debate. While Peretz [53,69] interprets a variety of data as evidence for more complex and specialized cognitive processing requirements than previously thought, and even modularity, pointing to a biological basis of musicality and some form of natural selection, Patel [70] argues that universality and processing specialization can be explained without evolutionary adaptation. Patel gives the example of the ability to make fire, which, although an invention, ‘extends deep into our species' past and is found in every human culture’ and ‘provides things that are universally valued by humans, including the ability to cook food, keep warm and see in dark places' [70, p. 46]. He also highlights the example of reading and writing—both cultural inventions—which are each partially associated with functional specializations in specific brain regions (product of neural plasticity) and in which disorders may sometimes be driven by genetic causes, at least for reading. However, musicality (unlike making fire, reading, writing or even music) is not a behaviour *per se* but a capacity that seems not to be taught or learned and appears to be present since early infancy [71–74], or even before delivery [29,30]. Thus, the question of whether music is an adaptation could be a dead-end (see [75]), but the origin of musicality is anything but.

## 3. The evolutionary study of music

Over more than two decades, researchers have focused their attention on the evolution of music, producing a great variety of evolutionary theories that range from Pinker's controversial description of music as *evolutionary cheesecake* [76] to purely adaptationist views [77,78]. Because these ideas have been reviewed and discussed elsewhere (e.g. [6,79,80]), we will not examine them in depth. Instead, this section addresses: (i) issues in the evolutionary study of music and (ii) some major ideas in the evolutionary theories of music, enabling us, in §4, to propose a theoretical model for the evolution of musicality.

### (a) Difficulties in the evolutionary study of music

Besides the fact that music does not seem to play an obvious direct role of biological relevance for survival and reproduction, the evolutionary study of music must face the problem that musicality is likely to consist of different, relatively independent components. Strong evidence for this can be found in the cases in which a disorder affects either pitch or rhythm processing, but not both (for reviews, see [53,66,81,82]), indicating the independence of these modules. This means, as Fitch [6, p. 174] points out, that different components of musicality might have followed independent evolutionary paths, and that ‘questions like “When did music evolve?” or “What is music for?” seem unlikely to have simple unitary answers'.

In addition, Justus & Hutsler [83] suggest that the evolutionary study of music might have been somewhat biased, favouring explanations based on natural selection over those involving cultural transmission. This is because most of the recent studies of the origins of music have been based on the approach of evolutionary psychology. This has required researchers to define criteria to assess whether music emerged as an adaptation (i.e. limited by innate factors, domain specificity, and conferring survival or reproductive advantages), or as an exaptation [83–85]. The problem, however, is yet more complex, as musicality (being a higher level cognitive domain such as language) probably involves both exaptations and adaptations, making the limits between them quite vague [83,85].

To eventually obtain a complete picture of the evolution of music, both biological (e.g. cognition, mother–infant interactions) and cultural (e.g. learned aesthetic preferences) aspects should be considered. However, these are so intimately connected in any musical manifestation or its perception that disentangling them is challenging. One possible solution is to study infants, assuming them to be individuals who have a very limited cultural experience. They can then be compared with adults to infer what does or does not require previous experience. This approach has provided important answers [71–74], but could intrinsically favour hypotheses related to the evolution of musicality from a parent–infant perspective.

### (b) Key ideas in evolutionary theories of music

Evolutionary theories of music are often linked to those of language. There are at least two main aspects that can be discussed separately: (i) the link between animal precursors and human music and language channels, and (ii) the evolution of human acoustic communication (including music and language).

#### (i) Animal precursors

Non-human acoustic communication has been compared to both language and music. In fact, vocalizations from many species are often called songs, because of their complexity and because they are learned [6]. However, except for gibbons, tarsiers, indri and perhaps also marmosets, tamarins and titi monkeys (see e.g. [86,87]), these complex song-like vocalizations occur only in birds and non-primate mammals such as cetaceans, suggesting that they do not share a common evolutionary path with music, or any other learned, complex human acoustic signals like language (but see e.g. [88]). There are, however, certain similarities and potential instances of convergent evolution that can provide models for the evolution of human acoustic communication (see [6,89]). Vocal learning, for example, seems to work in an analogous way in songbirds and humans, including the prevalence of local dialects and apparent individual differences [90]. In fact, bird brain areas involved in vocal learning have been compared to Broca and Wernicke regions of the human brain, as they activate when a bird hears or sings a song [91,92].

Furthermore, there are interesting parallels between human music and language and vocal signals of other animal species, particularly in instances where animal vocalizations have semantic- and syntax-like elements (for reviews, see e.g. [93–95]). Interesting cases of semantic-like elements (i.e. calls that have symbolic functions) come from domestic chickens, *Gallus domesticus*. Marler and colleagues [96,97], for example, showed that food calls produced by males are dependent on the quality of the food, and that females respond selectively to these calls. Furthermore, males are sensitive to the audience, producing significantly fewer calls when a rival male is present, and males are more likely to produce dishonest calls (i.e. in the absence of food) when females are further away than when they are nearby [98,99]. Similar sensitivity to social contexts and audiences has been shown for alarm calls in red junglefowl, *Gallus gallus* [100], which has different calls for different types of predator [101,102].

Perhaps some of the most interesting cases of semantic-like elements in non-human vocal communication, because of phylogenetic proximity with humans, are instances of vocalizations with some degree of symbolic content in primates. For example, vervet monkeys, *Cercopithecus aethiops*, like chickens, have different calls for different types of predators. The presence of jumping, flying and terrestrial predators (like leopards, eagles and pythons) is communicated through different calls, to which individuals respond differently: run into trees, look up or look down, respectively [103]. These distinct calls are evidence of effective categorization of other species, which individuals progressively develop with age and experience. Calls with symbolic functions have also been documented in other primate species [104–107].

Although seemingly simpler in nature, an interesting phenomenon of recombination of vocal elements has been documented in greater spot-nosed monkeys, *Cercopithecus nictitans.* This example is of particular interest because of the semantic-like properties that call series acquire by the recombination of different alarm calls depending on external events [108,109], thus involving both syntactic- and semantic-like properties.

Syntax-like elements have been widely studied and are usually present in species that produce vocalizations that are categorized as songs. Marler [95] divided syntactic elements into two types: (i) phonological syntax (or phonocoding), which is based on the recombination of individual, small phonetic units lacking meaning (e.g. phonemes in human language) to create sequences (e.g. words); and (ii) lexical syntax (or lexicoding), in which sequences are recombined to create strings (e.g. sentences) which have meaning at both the sequence (word) and string (sentence) level. While there are large differences in complexity between human and non-human examples, some animal vocalizations have structures that are like those of human language and particularly music, because of the absence of symbolic meaning.

In birds, some species have individual song repertoires with a complexity that exceeds that of non-human primates, and that are based on the recombination of elements (for a review, see [6]). Swamp sparrows, *Melospiza georgiana*, for example, have songs that consist of short individual, independent units, which are recombined into different sequences [110]. In winter wrens, *Troglodytes troglodytes*, each male has a repertoire of around 20 songs that incorporate and transform sequences of other winter wren songs [111,112], in a manner that seems to follow a flexible set of rules [113].

A different example of a potential animal precursor is entrainment, the synchronization to external rhythms, which is a phenomenon central to rhythm processing, musicality and perhaps speech (for a review, see [114]). Entrainment has been experimentally confirmed in at least one individual from another species, a sulfur-crested cockatoo, *Cacatua galerita eleonora* [115,116], and there is evidence of similar behaviour in other species, mainly parrots [117–119] and, importantly, chimpanzees [120–122].

Among mammals, however, the most complex vocal behaviour seems to be that of some cetaceans, particularly humpback whales, *Megaptera novaeangliae*, whose songs have syntactic elements analogous to those of songbirds. They are composed of units (analogous to phonemes), which are combined into phrases (relatively fixed sequences of units), and these into themes, which are a collection of phrases (including repetitions and combinations of phrases), which in turn are mixed to form songs with an average duration of 12 to 15 min [123]. Furthermore, humpback whale phrases and songs are constantly changing [124]. Like language and music in human cultures, this creates diversification between populations (e.g. [125,126]). Interestingly, the extent and rate of these changes seem to be motivated by novelty [127], and perhaps other factors. Similar changes in individual and population preferences are seen in songbirds (e.g. [128]; for a review, see [129]).

#### (ii) The evolution of human acoustic communication

Probably the best-known theory for the evolution of music, and one that proposes an adaptive function, is that music plays a role in sexual selection [18,78]. For example, the role that birdsongs play in mate choice seems akin to serenading in human societies. Musical ability may also be attractive in itself [130]. Although Fitch [89] highlights that there are no studies showing a positive relation between musical skills and reproductive success or offspring survival, recent studies have provided the empirical support for a theory of the evolution of music through sexual selection: for example, women prefer composers of more complex music around ovulation ([131–133]); there is a general increase in perceived mate value according to musical performance quality [134], and there is evidence of genes associated with both musical perception and production (reviewed in [135]).

However, music is in no way limited to mating contexts. This may indicate different evolutionary origins. For example, music seems to play an important role in promoting synchronization and cooperation, as well as group cohesion and identity (see [136]). Because of these social influences, Brown [77] suggests that music may have co-evolved with collective rituals, which could explain the universal association between music and rituals, as well as the rewarding properties of music from a psychological perspective. According to him, based on the capacity of music to promote social cohesion, and because music is overwhelmingly a social phenomenon, the survival value of music is not apparent at an individual, but only a group, level.

A somewhat similar hypothesis is that language evolved as a form of ‘vocal grooming’, to maintain social bonds in increasingly large groups [137–139]. In fact, when phylogenetically controlled, vocal repertoire size strongly predicts group size as well as grooming time in non-human primates [140]. Social bonding is maintained primarily via grooming in primates, but in increasingly large groups this behaviour, which tends to be a one-to-one activity, is less effective. While this theory is presented in relation to the origins of language, it suggests a stage of communal chorusing, lacking propositional meaning, which replaced grooming. Dunbar's theory of vocal grooming is consistent with archaeological evidence, as well as relationships between social group size and neocortex size ([137–139], see also [141]).

Dunbar's hypothesis resembles that of Darwin [18], who suggested a stage of vocal communication in human evolution more closely related to music (singing/humming) than to spoken language. If this is true, music could be a *fossil* of that hypothetical early stage of vocal communication among hominins, often referred to as musical protolanguage (e.g. [142,143]) or music-like protolanguage (e.g. [6]). This idea of a shared *common ancestor* between music and language is probably the most recurrent idea in evolutionary musicology. Similar models covering protolanguage stages that relied on musical or music-like elements have been proposed (e.g. [17]), including Brown's musilanguage model ([144], see also [145]), which suggests the idea of an expression spectrum, in which purely referential meaning (lacking emotional content) lies at one end and purely emotional meaning at the other. The main strength of these models, beyond potentially addressing the origins of both music and language, is that they could explain the complex similarities between these (see §2b).

Trehub [73] and Dissanayake [146] have suggested that the primary role of music, and songs in particular, is to aid parent–infant communication. This hypothesis is supported by the apparent universality of lullabies ([34], see also [36–38]), and their calming effects on infants, commonly used to aid them to fall asleep. This idea is also compatible with the existence of IDS and its prevalence in parent–infant interactions; IDS has characteristic vocal modulation patterns that are detected by infants [38,147] and has important effects on language learning [148], communicating affect [149] and strengthening of mother–infant bonds, which could indicate that IDS is an important component in the development of musicality ([73,146], see also [150–152]). Furthermore, this hypothesis is compatible with Dunbar's hypothesis of vocal grooming [137–139], and Falk [7,14] suggested that IDS could be a precursor of the social grooming stage that may have led to language.

In comparison with a sexual selection hypothesis for the origin of musicality, a theory based on parent–infant interactions appears to have important advantages. As pointed out by Fitch [89], it can explain the early development of musical perception abilities, as well as the universal existence and effects of lullabies and IDS, therefore providing hints for a model that could explain, not only music and language, but also IDS.

To summarize, all evolutionary theories about musical capacities share an important component of emotional cohesion, invoking benefits obtained during some form of social interaction, whether in mate choice, social bonding within groups, or parent–infant interaction. While evolutionary theories tend to view musicality as a product of one or other of these selection pressures, it is important to contemplate the possibility that different kinds of benefits for musicality may have been in play at different times during its evolution.

#### (iii) Alternative views

The two previous sections outlined several lines of evidence, based on which we will propose a new model for the evolution of musicality. But before we turn to that task, we should first point out criticisms of some aspects of this evidence. First, it is essential to mention that correlations among traits do not imply adaptations, and that this is an important limitation of several theories (for a detailed discussion, see [153]).

In addition, it is important to consider the genuine possibility that music-like behaviours in non-human animals may be a be poor analogue of human music, especially when comparing it with behaviours and cognitive processes of distant species (perhaps excluding rhythm as, for example, thrush nightingales, *Luscinia luscinia*, have been shown to have universal rhythm categories that are composed of patterns strikingly similar to those of music [154]). In fact, even the perceptual phenomena in (at least some) non-human animals could have very little in common with music. For example, work with European starlings, *Sturnus vulgaris*, has shown that auditory perception in those songbirds vastly differs from that of humans ([155]; see also [156]) and that, unlike in humans, sound pattern recognition is based on absolute spectral shapes rather than pitch [157].

Furthermore, despite the prevalence of arguments in support of a sexual selection hypothesis, some of the supporting evidence has been called into question (for a review, see [158]). As highlighted by Mosing *et al*. [159], the sexual selection hypothesis makes several specific predictions: (i) skilled musicians should have greater mating success than less musically skilled individuals; (ii) musical ability should indicate genetic quality and, therefore, there should be an association between musical ability and other putative traits related to genetic quality; and (iii) there must be at least partial overlap in the genetic influences in the associations between the first two predictions. In a sample of over 10 000 twins, Mosing *et al*. found little support for these predictions. Remarkably, they in fact found that musical ability was negatively associated with measures of mating success.

Likewise, in a recent theory for the evolution of music proposed by Mehr *et al*. [153], the authors pointed to the various weaknesses of a hypothesis of music as a signal of mate quality. In particular, a hypothesis based on a role of music as a signal of male mate quality—which would have co-evolved with female preferences, similar to other species—predicts that sexually dimorphic signals in the form of music should be emphasized in men. However, there is little evidence of sex differences in musicality (see [153]), and, in fact, women may produce more novel songs than men [160].

Similarly, Dunbar's hypothesis of vocal grooming [137–139,161], which is based on a positive correlation between group size and grooming time, has also been heavily criticized in recent years [162,163]. Grueter *et al*. [162] argue that the association between group size and grooming time is confounded with substrate: they contend that grooming time should be higher in terrestrial (versus arboreal) species, given higher exposure to ectoparasites. They found that terrestriality predicts grooming time better than group size. While these analyses were disputed by Dunbar & Lehmann [164], newer criticism of the vocal grooming hypothesis was presented by Jaeggi *et al*. [163]; in their analyses, terrestriality was again found to be an important predictor of grooming time, and no evidence of vocal grooming as a less time-consuming, more efficient form of bonding (as stated in the original form of the vocal grooming hypothesis) was found.

Despite these criticisms, the bulk of evidence suggests that there are at least close parallels between forms of vocal communication in human and (some) non-human species, suggesting at least some form of similarity in the selection pressures that have led to similar solutions. In addition, the evidence suggests that music—and particularly rhythm—plays an important role in interactions within human groups.

## 4. Towards a model for the evolution of musicality

As pointed above, we believe evolutionary theories related to this subject should not focus on music primarily, but on the ability to process musical information (musicality), understanding the cognitive components and potential modules of musicality, and studying their evolutionary history by tracking their role in several domains. It is important to consider that modularity (i.e. organization into independent components that interact with other components) does not equal domain specificity [53] and, if music, language and perhaps IDS have a common evolutionary history, then musicality (or components of it) might not be limited to music processing and could in fact play a role in other domains. For example, new evidence suggests that musicality affects the perception and imitation of nuanced pronunciations in languages [42].

Musicality seems to integrate several processing modules, for at least two of which there is common agreement: pitch and rhythm processing [53]. Importantly, most previous models of music evolution do not treat these two components separately (for a notable exception, see [153]). However, owing to their relative independence, they may have had separate evolutionary origins and have been shaped under independent evolutionary processes [6], as discussed in §3a.

### (a) Developing the model

As most theories for the origins of music suggest, music and language could be *descendants* of an earlier, vanished form of vocal communication among ancestral hominin species (§3b(ii)). This could help to explain the relationship between music and language (§2b) and, potentially, IDS. Among these theories, a model based on the role of musicality in infant–parent communication has particular strengths, as it could further explain the universal features of IDS and lullabies, as well as the musicality of babies, not dependent on previous experience ([83,84] see also [85]).

The most challenging issue for any model, however, is to explain how human populations changed from a state where musicality, and its components, was non-existent or very modest towards one where they become fully developed. Here, we argue that selection on parent–infant communication could have driven such change, because human infants are so vulnerable that even small improvements in communication and bonding might make the difference between life and death. Indeed, the same argument has been made by researchers in a different sensory modality: Wyatt [165] suggests that secretions from the areolar glands on the breast may be the best place to focus in the ongoing search for a human pheromone, because of its potentially critical importance in facilitating successful suckling when the infant is most vulnerable.

Relative pitch (RP)—an important component of pitch processing and musicality [53]—is the ability to process pitches in relation to each other. Without this ability, individuals with different voice registers would be incapable of recognizing (or imitating) a melody as such when sung by, for example, children versus adult women or adult men, who tend to have different registers. RP tends to improve with practice [166,167] and may be better in people who speak tonal languages [168]. Across species, RP seems to be rare, but there is evidence of RP in songbirds like the black-capped chickadee, *Poecile atricapillus* [169] and European starlings, *Sturnus vulgaris* [170], and mammals like ferrets, *Mustela putorius furo* [171]. However, to our knowledge, there are no species that can match human RP abilities. Crucially, evidence shows that human neonates are able to process pitch intervals in a similar manner to adults [172]. Like Trainor *et al*. [173], we suggest that IDS primarily functions to communicate affective states, as supported by the preference of infants for IDS conveying positive over negative emotions [174,175].

Likewise, rhythm processing seems to be of particular importance in terms of synchronization and entrainment, even from infancy (e.g. [176,177]); for a musical entrainment review, see [178]. Today, rhythm processing is important in IDS, consistent with a model based on the role of musicality in infant–parent communication. However, the influence of music in synchronizing behaviours and promoting bonding (e.g. [179–182]) is consistent with hypotheses based on music playing a role in promoting group cohesion, as in Dunbar's theory of vocal grooming [137–139,161], or with hypotheses in which music functions as a credible signal for coalitional intent [153,183,184]. It is also manifest in group activities: for example, contemporary armies around the world march to music, and common analogous examples include rhymes and chants of sports fans and protesters. Neither is this a solely modern phenomenon: notable traditional examples include Zulu war chants and the *haka* from the Māori people of New Zealand. Music seems to reduce physical exertion [185] and increase pain threshold [181,182], which could also partially explain why music is common when human groups perform repetitive, physically demanding tasks.

In other words, rhythm processing seems to enable mechanisms for social bonding and group identification through synchronized behaviours, while pitch processing enables parent–infant bonding and communication through the inferral of emotional states [174,175], laying the ground for the active communication of those states in any form of protolanguage.

In general, the evidence is consistent with a model for the evolution of musicality based on its role in infant–parent communication. Communication between infants and parents is critical to survival: human children are born relatively underdeveloped, parental care is exceptionally long, and children require strong parental bonds to guarantee care and avoid potentially fatal neglect. In fact, IDS is associated with oxytocin levels and other neuropeptides involved in attachment mechanisms (e.g. [186–188]). Better parent–infant communication, and particularly mother–infant bonding, could facilitate social learning in infants, allowing them to acquire the necessary skills to survive [189]. Moreover, a developmental tool is necessary for language to evolve, and parent–infant communication is crucial in this aspect (as seen today in IDS, [190]).

If parent–infant communication promotes infant survival and development, then selection could have acted on individual variation in musicality to the benefit of those with better ability. Moreover, because musicality seems to be at least partially hereditary (see [67,135]), adults with a good level of musicality could produce offspring better equipped to process this information and with the potential of being yet more successful parents, adding a new level to the selection pressure for musicality. In fact, there are primate precursors of guided vocal learning at least in marmoset monkeys [191–193], providing grounds for selection to act upon.

Furthermore, this model could integrate the evidence in support for a sexual selection hypothesis, including the preference for composers of more complex music around ovulation [131,132,134,153]. If musicality affects infant survival, then cues of musicality would likely become sexually selected. Choosing a partner with musical abilities would be appealing and relevant if it implies a capacity to bond and empathize with infants and other members of the community, and to produce offspring with these abilities. This could explain the role of vocal modulation—such as an increase in *f*_0_ variability, analogous to that of IDS—during courtship [8,11] and its detection and preference by listeners [8].

Sexual selection would, however, require some display of musicality, which could have been manifested in a music-like protolanguage, and would exploit the capacity of music to coordinate behaviour and promote social bonding. In a society where basic forms of group chorusing (proto-songs?) start to appear in the context of social rhythmic and coordinated behaviours, the interaction between the voice of male adults and women or children would tend to create octaves and fifths [34], provided there is a perceptual preference for these consonances which in fact does not seem to be unique to humans (for a review, see [194]). These group activities would start to promote, not only social bonding, but also group identity.

The theoretical splitting between music and language from a common ancestor (music-like protolanguage) might have been a product of the increased relevance that propositional syntax [144] and semantics played in human communication, building an increasing specialization towards language. Nevertheless, language itself is of little use in the context of interactions with pre-linguistic infants, leaving a domain in which musicality remained essential, being uniquely able to communicate and influence emotional states. For this to occur, however, musicality needs to be present in both infants and adults, allowing the cognitive musical abilities to be employed for other purposes in which it remained influential (e.g. group cohesion and social identity).

**Figure 1.**
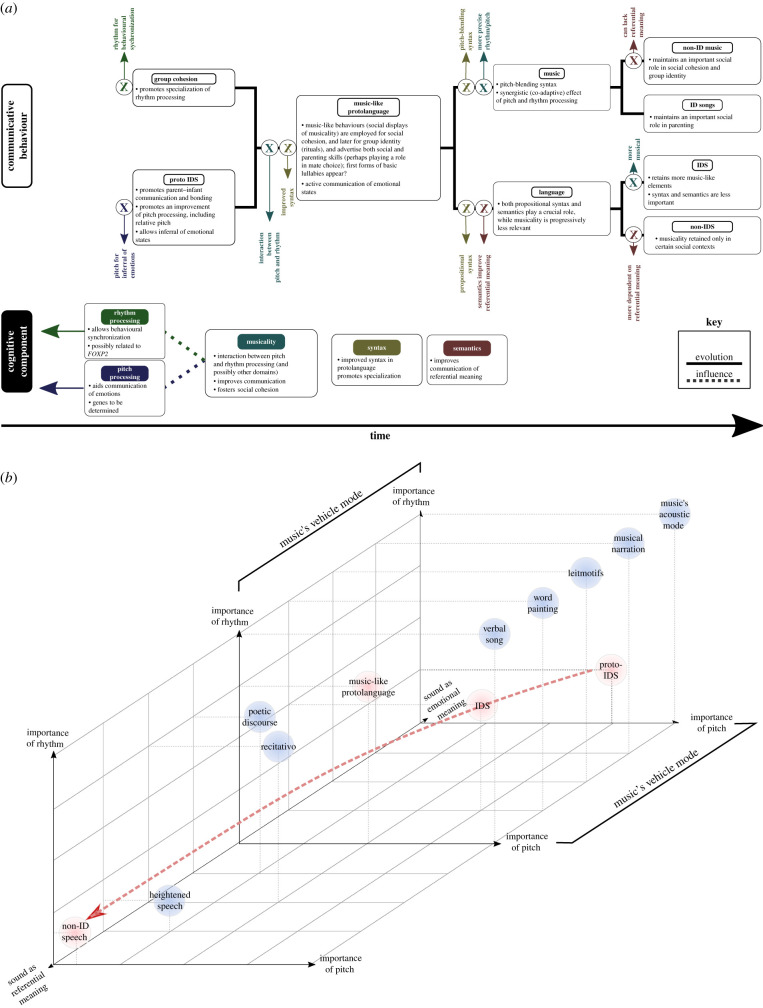
(*a*) Model for the evolution of musicality and its role in human vocal communication. Musicality is presented as a simplified convergence of pitch and rhythm processing, which promotes parent–infant communication and bonding, primarily infant-directed vocalizations (proto-IDS) through pitch, and behavioural synchronization through rhythm. Music exploits both pitch and rhythm in more precise manners than other forms of communication, and language depends more on propositional syntax and semantics, and less on precise rhythmic or melodic patterns. Coloured Xs represent influences that derive in specialization and different communicative behaviours. (*b*) Hypothetical location of different functions of human acoustic communication, following Brown's musilanguage model [144, Fig. 16.1]. In addition to Brown's original meaning axis (diagonal axis, from referential to emotional), the location of each function is located according to the importance of rhythm (vertical axis) and pitch (horizontal axis) in conveying such meaning. Blue circles represent functions included by Brown, while pink circles represent forms included in our model, but not present in the original musilanguage model. The red arrow represents the transition in meaning, from emotional to referential, of parent–infant communication, becoming more referential as the infant grows. Music forms are represented as having a highly important role of both pitch and rhythm, but this can obviously change. For example, in music that promotes group cohesion, like military marches, rhythm tends to have a fundamental role, while pitch's importance can vary widely, from melodic marches to exclusively rhythmic ones that lack any melody.

Although ancestral primate societies lived in groups, and social cohesion and bonding were likely achieved by other means (similar to other primates; e.g. [137–139]), parent–infant communication became fundamental because of increasing altriciality, and the resulting prolonged parental care and need to acquire more and more complex information via social learning. In short, we are suggesting that musicality is based on pre-existing abilities (see also [136]), that musicality itself pre-dates music, and that its primary and original purpose was not music (music being, in this view, an epiphenomenon). This model for the evolution of musicality is summarized in figure 1.

### (b) Implications and tests

If this model accurately portrays the evolution of musicality and its role in complex human vocal communication, musicality could be partially a *fossil* of our musical brain, whose original communicative purpose was bonding and communication between parents and infants, primarily through expressive pitch contours, and later the social communication of intentions and emotions (see [195] in part I, who shows how vocal cues of expressivity also drive the perception of emotion in music) to promote social bonding and coordination (music-like protolanguage) through an increased importance and precision of rhythm. It is important to emphasize the word *partially*, because the original purpose of aiding communication and bonding between parents and infants is still biologically relevant today in IDS and lullabies, as well as its power to promote group cohesion and social identity, evident today in ritual music, for example.

Moreover, the model incorporates selection pressures that promote the appearance of musicality-related abilities, including being able to navigate a complex social environment and develop strong social bonds, particularly those between mother and offspring (which also require offspring to be born relatively underdeveloped and dependent on their mothers or other adults to survive, incidentally common among many mammal and bird species). These pressures can be tested in other species according to the complexity of their vocal communication. In fact, there is evidence suggesting a positive association between social and vocal complexity in several species, including Carolina chickadees, *Poecile carolinensis* [196], sciurid rodents (marmots, *Marmota* spp.; prairie dogs, *Cynomys* spp.; ground squirrels, *Spermophilus* spp.) [197] as well as other ground-dwelling mammals [198] and bats of the suborder Microchiroptera [199]. However, use of species more closely related to humans (i.e. primates) might allow more specific predictions to be tested, regarding the evolutionary pressures that promote vocal complexity (like coevolution of social and vocal complexity, [140]), but also those that forged human musicality.

This model predicts that primate species living in particularly complex social environments are the most likely candidates to show musicality-like skills. Indeed, social complexity could be positively associated with more complex communicative systems [200,201], including both vocal diversity and flexibility (e.g. [202,203]; see also [153]). For example, bonobo (*Pan paniscus*) vocal interactions respect temporal rules like turn-taking, akin to those of human conversations [88]. Regarding infant–parent bonding, pygmy marmosets, *Cebuella pygmaea*, a socially complex species with cooperative breeding, have been shown to use a form of ‘babbling’ during infancy that decreases as they grow older [204], and such babbling in both infants and juveniles is associated with increased social interactions with other group members [205]. In fact, cooperative breeding has been argued to promote the evolution of language ([206,207]; see also [86]). However, perhaps the most interesting example comes from gelada baboons, *Theropithecus gelada*, a species that lives in a less complex ecological environment, but a more complex social environment, than the closely related chacma baboons, *Papio ursinus*, and has more vocal complexity [208]. Geladas, in fact, have been shown to synchronize their calls [209] and use both rhythm and *melody* in their vocal interactions [210].

In human language contexts, musicality seems to be required to perceive *f*_0_ contours, of which variability in fundamental frequency (*f*_0_ variability) modulation seems to be a good indicator [8–11,211]. While most evidence points toward an important role of *f*_0_ modulation in courtship contexts and mate choice, new lines of evidence have highlighted the importance of *f*_0_ variability modulation and other forms of musicality in more general contexts, including the recently reported association between *f*_0_ variability and cooperativeness [212], and the role of musicality in allowing individuals to capture the nuances of linguistic non-verbal aspects (e.g. [42,213]).

Recently, two noteworthy but opposing theories for the evolution of music have been proposed. The model put forward by Savage *et al*. [141] has social bonding as its main function. By contrast, Mehr *et al*. [153] propose that music functions as a credible signal for both coalitional interactions (i.e. signalling coalition strength, size and coordination ability) and parental attentiveness, functions that evolved from territorial advertisements and contact calls, respectively. In addition, they provide important criticisms of the dominant views of music evolution—that is, that music evolved through sexual selection, to promote social bonding, or as a by-product of other adaptations.

Our model shares some features with both these ideas, but also has some key differences. We fully share the view of Savage *et al*. of the importance of social bonding, but we believe that music, and especially musicality, need to be placed in a field where communication is much wider than simply music. Furthermore, in contrast with Savage and colleagues, we differentiate the evolution of pitch- and rhythm-related cognition, while their model better fits rhythm only. We also agree with Mehr and colleagues that sexual selection is unlikely to be the primary driver of human musicality, that melody evolved in the context of parent–offspring communication and that rhythm evolved in the context of group interactions. However, if musicality is important for creating and maintaining bonds with infants, or as a signal of parental attention and care [153], cues of this ability may exist in species with alloparenting and/or biparental care, and hence be preferred in potential long-term partners. In humans, this would likely be in the form of voice modulations akin to those of IDS. This would imply that those cues are present in both males and females, as opposed to preferably in males, as a traditional mate quality hypothesis would imply. Thus, our model makes an array of unique predictions that can be bold, broad or specific:

(i) Musicality should be associated with better parental abilities and/or disposition, rather than increased mating success.
(ii) Given its importance in several different social contexts, in both infancy and adulthood, we may not observe any large pubertal change in the importance of musicality, despite normally seeing such changes in other sexually selected traits.
(iii) Musicality itself should not be sexually dimorphic, but sex differences in musical performance, particularly in mating-relevant contexts, could exist. It is important to consider that not all sexually selected traits must be sexually dimorphic, particularly in species with mutual mate choice (like preferences for some personality traits such as agreeableness (e.g. [214]).
(iv) In the light of alloparenting in humans, because parents are rarely the only caretakers of infants, cues of musicality from potential caretakers may be important as a sign of trust and ability to bond with, and care for, infants.
(v) A core characteristic of IDS (and in fact, ‘infant-directedness'), as a specific but universal form of vocal modulation, is increased pitch variability (e.g. [38]). Thus, infants should prefer samples of IDS with experimentally increased pitch variability over unmanipulated ones.
(vi) In courtship contexts, pitch variability should correlate with the perceived attractiveness of the target listener and should be a stronger predictor than mean voice pitch (which has been argued to be a cue of underlying mate quality (e.g. [215]). Preliminary evidence already supports this prediction [11], but it should be especially clear in prospective committed relationships.
(vii) If musicality is cued in non-musical contexts by changes in pitch variability, as indicated in courtship studies of voice modulation (e.g. [8–11]), then the ability to detect these subtle modulations should be dependent on pitch discrimination skills; any potential benefit of such vocal modulations would be limited by the listener's capacity to perceive such changes.
(viii) In couples with children, pitch discrimination skills of each parent should be positively associated with satisfaction with their partner as a mother or father.
(ix) Musical rhythm processing should evolve only in species with need for coordination (e.g. cooperation).
(x) Musical pitch processing should evolve only in species with altriciality and long parental care, where infants' survival chances are linked to the strength of their bonds and ability to communicate with their parents and/or other caregivers.
(xi) Musicality (and perhaps some musical manifestations) should evolve mainly in species with the requirements for predictions (ix) and (x).

The model proposed here is relatively simple, as it is based only on two potential cognitive modules (i.e. pitch and rhythm processing, corresponding to the two principal components found by Mehr *et al*. [39]), but it provides a general view consistent with the most recent evidence and could explain the range of human complex vocal communication. It shares important aspects with previous models, including the idea of a common ancestor to both music and language. However, it also incorporates several novel aspects, in particular: (i) that models' focus should be on musicality rather than music; (ii) that modules of musicality, like pitch and rhythm processing, may have different evolutionary paths and could have been shaped by different selection pressures; (iii) that musicality may pre-date music itself; and (iv) that musicality could play a role today, not only in music, but also in IDS, and at least certain language contexts like courtship. In addition, we believe that infant–parent bonding is the strongest candidate to explain the emergence of musicality in the human lineage. It might be more than a link between music and language: it could be the very purpose of musicality.

## Data accessibility

This article has no additional data
